# Supplementary material for: A multiplexed bacterial two-hybrid for rapid characterization of protein–protein interactions and iterative protein design
Source: Nat Commun. 2023 Aug 2;14:4636. doi: 10.1038/s41467-023-38697-x (PMC10397247; doi:10.1038/s41467-023-38697-x)
Supplement: Supplementary file 2 — Description of Additional Supplementary Files [file 41467_2023_38697_MOESM2_ESM.pdf]

**Title: Supplementary Data 1**
**Description: OLS oligonucleotides for gene synthesis of libraries**
The table contains the DNA sequences of all ordered oligonucleotides for all libraries (CC0, CC1, CCNG1 and CCMax).

**Title: Supplementary Data 2**
**Description: Sequences of proteins used in libraries**
The table contains all the coiled-coils sequences tested in all orthogonal sets.

**Title: Supplementary Data 3**
**Description: Oligonucleotides for PCR**
The table contains all the oligonucleotides used in the project.

**Title: Supplementary Data 4**
**Description: Plasmids referred to by name**
The table contains all the plasmids used in the project.

**Title: Supplementary Data 5**
**Description: Interaction Scores from all libraries**
The table contains all the interactions scores measured form all libraries.

**Title: Supplementary Data 6**
**Description: Largest orthogonal subset for each set in the CCMax library**
The table contains the experimentally identified orthogonal sets, identified at different orthogonality gaps.

**Title: Supplementary Data 7**
**Description: Largest orthogonality gaps by number of on-target interactions**
This table contains the best sets – i.e. the largest ones with the largest experimental orthogonality gaps.

**Title: Supplementary Data 8**
**Description: Comparisons between libraries**
Describes the libraries, the sizes, algorithms used in design and the largest sets identified.

**Title: Supplementary Data 9**
**Description: Uncertainty analysis**
Describes the analysis that was used to determine a value for the orthogonality gap. Standard deviation and Median Absolute Deviation of difference in interaction score between two technical repeats of the same library, CC0, as well as same pairs with different orientation of split enzyme were analyzed.
