# Supplementary material for: A multiplexed bacterial two-hybrid for rapid characterization of protein–protein interactions and iterative protein design
Source: Nat Commun. 2023 Aug 2;14:4636. doi: 10.1038/s41467-023-38697-x (PMC10397247; doi:10.1038/s41467-023-38697-x)
Supplement: Supplementary file 14 — Source Data [file 41467_2023_38697_MOESM14_ESM.zip › Supplementary File S1 Visualization of designed sets.zip!/06_set_visualizations/03_extra_html/MODEL_basic-rep-nter_core-Ridge-W10.html]

08\_aligned\_fit\_iterative


In [1]:

```
from __future__ import division, absolute_import, print_function
%load_ext autoreload
%autoreload 2

import os 
print(os.getcwd()) 
    
#import hepran
#import hepran.bzipscore as bz
#import hepran.bcipa as bc
#import hepran.utils as u
#import hepran.registers as r
#import hepran.agadir as ag

import pandas as pd
import numpy as np
import seaborn as sns
    
%matplotlib inline

import matplotlib as mpl
import matplotlib.pylab as plt

import sklearn as sk


CCO = os.environ.get("CCO",r"C:\Projekti_KI\ortoCC\CoiledCoilOrtho")

from score_utils import *
```

```
d:\data\ortoCC\fine_tune_scoring_function
```

In [2]:

```
from interactive_set_plot import *

import bokeh
import bokeh.resources
import bokeh.plotting as bp
from bokeh.models import HoverTool
from IPython.core.display import display, HTML
import hepran
bp.output_notebook()

import sklearn
from sklearn import linear_model
from sklearn.externals import joblib

from IPython.display import display, HTML
```

Loading BokehJS ...

In [3]:

```
%%time
df = pd.read_excel('data/round0_fiting.xlsx',index_col=[0,1])
df = df.apply(get_CC_features, axis=1)
```

```
Wall time: 1.09 s
```

In [4]:

```
fit_metrics_all =  get_metrics_df().set_index('N_iter fit_type fit_class'.split())
model_features_all = get_features_df().set_index("N_iter fit_type".split())
```

In [5]:

```
def set_weights(name, df):
    
    df['weights'] = 1
    if name == "W10L15H55":
        df['weights'] = 1
        df.loc[df.Tm < 15, 'weights'] = 10
        df.loc[df.Tm > 55, 'weights'] = 10
        df.loc[df.on_target, 'weights'] = 10
    if name == "W10L15H50":
        df['weights'] = 1
        df.loc[df.Tm < 15, 'weights'] = 10
        df.loc[df.Tm > 50, 'weights'] = 10
        df.loc[df.on_target, 'weights'] = 10
    if name == "W10":
        df.loc[df.on_target, 'weights'] = 10
    if name == "W10L":
        df['weights'] = 1
        df.loc[df.Tm < 15, 'weights'] = 10
        df.loc[df.on_target, 'weights'] = 10
```

In [6]:

```
basic = 'c_NN c_IN c_II es_EE es_EK es_KK'.split()
basicL = 'c_NN c_IN c_II es_EE es_EK es_KK cd_LL'.split()
basicCP = 'cp_EEIIEE cp_EEINEE cp_EENNEE cp_EKIIEK cp_EKINEK cp_EKNIEK cp_EKNNEK cp_KKIIKK cp_KKINKK cp_KKNNKK'.split()
nterm_core = 'nterm_c_NN nterm_c_IN nterm_c_II'.split()
cterm_core = 'cterm_c_NN cterm_c_IN cterm_c_II'.split()
core_ends = nterm_core + cterm_core 

core_vertical = 'cv_NN cv_II cv_NI cv_IN'.split()
es_vertical = 'esv_KK esv_EE esv_EK esv_KE'.split()

core_t_vertical = 'cvt_INI cvt_IIN cvt_NII cvt_NNI cvt_NNN cvt_NIN cvt_INN cvt_III'.split()
es_t_vertical = 'esvt_EKK esvt_EEK esvt_EEE esvt_EKE esvt_KEE esvt_KKK esvt_KKE esvt_KEK'.split()
interface_rep = ['interface_repulsion']

fit_fields_dic = {
    "basic": basic,
    "basic-ends": basic + core_ends,
    "basic-rep": basic + interface_rep,
    "basic-core_vertical": basic + core_vertical, 
    "basic-ends_rep": basic + core_ends + interface_rep,
    "basic-rep-core_vertical": basic + interface_rep + core_vertical, 
    "basic-rep-nter_core-vertical_t_all": basic + interface_rep + nterm_core + core_t_vertical + es_t_vertical,
    "basic-rep-core_t_vertical": basic + interface_rep +  core_t_vertical,
    "basic-rep-nter_core-core_vertical": basic + interface_rep + nterm_core + core_vertical,
    "basic-rep-core_vertical": basic + interface_rep + core_vertical,
    "basic-rep-es_t_vertical": basic + interface_rep + es_t_vertical,
    "basic-rep-core_vertical": basic + interface_rep +  core_vertical,
    "basic-rep-nter_core": basic + interface_rep + nterm_core,
    "basicL": basicL,
    "basicL-ends": basicL + core_ends,
    "basicL-rep": basicL + interface_rep,
    "basicL-core_vertical": basicL + core_vertical, 
    "basicL-ends_rep": basicL + core_ends + interface_rep,
    "basicL-rep-core_vertical": basicL + interface_rep + core_vertical, 
    "basicL-rep-nter_core-vertical_t_all": basicL + interface_rep + nterm_core + core_t_vertical + es_t_vertical,
    "basicL-rep-core_t_vertical": basicL + interface_rep +  core_t_vertical,
    "basicL-rep-nter_core-core_vertical": basicL + interface_rep + nterm_core + core_vertical,
    "basicL-rep-core_vertical": basicL + interface_rep + core_vertical,
    "basicL-rep-es_t_vertical": basicL + interface_rep + es_t_vertical,
    "basicL-rep-core_vertical": basicL + interface_rep +  core_vertical,
    "basicL-rep-nter_core": basicL + interface_rep + nterm_core,
    "basicL-nter_core": basicL + nterm_core,    
    "basicCP": basicCP,
    "basicCP-ends": basicCP + core_ends,
    "basicCP-rep": basicCP + interface_rep,
    "basicCP-core_vertical": basicCP + core_vertical, 
    "basicCP-ends_rep": basicCP + core_ends + interface_rep,
    "basicCP-rep-core_vertical": basicCP + interface_rep + core_vertical, 
    "basicCP-rep-nter_core-vertical_t_all": basicCP + interface_rep + nterm_core + core_t_vertical + es_t_vertical,
    "basicCP-rep-core_t_vertical": basicCP + interface_rep +  core_t_vertical,
    "basicCP-rep-nter_core-core_vertical": basicCP + interface_rep + nterm_core + core_vertical,
    "basicCP-rep-core_vertical": basicCP + interface_rep + core_vertical,
    "basicCP-rep-es_t_vertical": basicCP + interface_rep + es_t_vertical,
    "basicCP-rep-core_vertical": basicCP + interface_rep +  core_vertical,
    "basicCP-rep-nter_core": basicCP + interface_rep + nterm_core,
}
extra_cols=[]
```

In [7]:

```
fit_type_string = "basicCP-rep-nter_core"
lm_type = "BayesianRidge" #Ridge, ElasticNet, SGDRegressor, BayesianRidge
weight_string="W1" #W1 W10 W10L15H50 W10L15H55

#read ENV vars if they are set
fit_type_string = os.environ.get('fit_type_string', fit_type_string)
lm_type = os.environ.get('lm_type', lm_type)
weight_string = os.environ.get('weight_string', weight_string)
```

In [8]:

```
fit_type = fit_type_string
fit_fields = fit_fields_dic[fit_type_string]

set_weights(weight_string, df)
if weight_string:
    fit_type += "-" + weight_string


Q=df
N_iter = 0
print("Iteration: ", N_iter)
print("Num points: ", len(Q))
lm, R2 = make_model('Tm', fit_fields, Q, lm_type)
fit_type += "-" + lm_type 

df['score'] = lm.predict(df[fit_fields])
df['pos'] = 0


df = df.apply(get_formated_seq, axis=1)
df['IDs']=df.index
title = str(N_iter)+"_"+fit_type
#bp.output_file(title=title, filename='models\\{title}.plot.html'.format(**locals()))
p = draw_scatter_interactive('Tm','score',df, y_range=(0,80),                                
                                 title=title, save_to_file=False)


bp.show(p)

mpl_plot_fit(title, df)

fit_metric = get_FIT_dataframe(Q, lm, N_iter, fit_type, N_feat=len(lm.coef_)+1, N_samples=len(Q))
fit_metrics_all = fit_metrics_all.append(fit_metric)
display(fit_metric)
model_features = get_model_features(lm, fit_fields, N_iter, fit_type)
model_features_all = model_features_all.append(model_features)
display(model_features)


joblib.dump(lm, 'models/{title}.model'.format(**locals())); 
df.to_excel('models/{title}.score.xlsx'.format(**locals()));
bp.save(p, title=title, filename='models\\{title}.plot.html'.format(**locals()), resources=bokeh.resources.INLINE);
```

```
Iteration:  0
Num points:  172
```

|  |  |  | corrR | R2\_score | RMSE | med\_abs\_err | explained\_var | Baysian\_IC | Akaike\_IC | N\_samples | N\_feat |
| --- | --- | --- | --- | --- | --- | --- | --- | --- | --- | --- | --- |
| N\_iter | fit\_type | fit\_class |  |  |  |  |  |  |  |  |  |
| 0 | basic-rep-nter\_core-W10-Ridge | all | 0.699908 | 0.782343 | 10.893895 | 2.811030 | 0.466846 | 878.164125 | 1728.910736 | 172 | 11 |
|  |  | Tm>25 | 0.671339 | 0.677368 | 9.294077 | 2.737149 | 0.317134 | 473.674344 | 878.200003 | 95 | 11 |
|  |  | Tm>55 | 0.766732 | -0.849728 | 6.377983 | 2.376602 | -0.011735 | 80.909484 | 110.826656 | 14 | 11 |
|  |  | on\_target | 0.474465 | -0.062665 | 6.393989 | 2.328906 | 0.069435 | 71.862572 | 96.347479 | 12 | 11 |

|  |  | feature | coef |
| --- | --- | --- | --- |
| N\_iter | fit\_type |  |  |
| 0 | basic-rep-nter\_core-W10-Ridge | c\_NN | 1.787093 |
|  | basic-rep-nter\_core-W10-Ridge | c\_IN | -3.574187 |
|  | basic-rep-nter\_core-W10-Ridge | c\_II | 1.787093 |
|  | basic-rep-nter\_core-W10-Ridge | es\_EE | -2.597351 |
|  | basic-rep-nter\_core-W10-Ridge | es\_EK | 3.080572 |
|  | basic-rep-nter\_core-W10-Ridge | es\_KK | -0.483221 |
|  | basic-rep-nter\_core-W10-Ridge | interface\_repulsion | -0.316875 |
|  | basic-rep-nter\_core-W10-Ridge | nterm\_c\_NN | 3.481198 |
|  | basic-rep-nter\_core-W10-Ridge | nterm\_c\_IN | 1.399977 |
|  | basic-rep-nter\_core-W10-Ridge | nterm\_c\_II | -4.881175 |

In [9]:

```
N_iter = 1
seqs = df

%run -i 08_aligned_fit_DO_ITER.py
```

|  |  |  | corrR | R2\_score | RMSE | med\_abs\_err | explained\_var | Baysian\_IC | Akaike\_IC | N\_samples | N\_feat |
| --- | --- | --- | --- | --- | --- | --- | --- | --- | --- | --- | --- |
| N\_iter | fit\_type | fit\_class |  |  |  |  |  |  |  |  |  |
| 1 | basic-rep-nter\_core-W10-Ridge | all | 0.794191 | 0.845300 | 9.184224 | 2.621092 | 0.625250 | 819.438059 | 1670.184670 | 172 | 11 |
|  |  | Tm>25 | 0.764343 | 0.748369 | 8.207951 | 2.441750 | 0.494007 | 450.062284 | 854.587942 | 95 | 11 |
|  |  | Tm>55 | 0.794867 | -0.503175 | 5.749555 | 2.289025 | -0.618444 | 78.005059 | 107.922231 | 14 | 11 |
|  |  | on\_target | 0.473920 | 0.149498 | 5.720202 | 2.288988 | 0.177541 | 69.190073 | 93.674980 | 12 | 11 |

|  |  | feature | coef |
| --- | --- | --- | --- |
| N\_iter | fit\_type |  |  |
| 1 | basic-rep-nter\_core-W10-Ridge | c\_NN | 0.984456 |
|  | basic-rep-nter\_core-W10-Ridge | c\_IN | -4.327710 |
|  | basic-rep-nter\_core-W10-Ridge | c\_II | 7.318866 |
|  | basic-rep-nter\_core-W10-Ridge | es\_EE | -0.211516 |
|  | basic-rep-nter\_core-W10-Ridge | es\_EK | 6.112698 |
|  | basic-rep-nter\_core-W10-Ridge | es\_KK | 2.050041 |
|  | basic-rep-nter\_core-W10-Ridge | interface\_repulsion | -0.250022 |
|  | basic-rep-nter\_core-W10-Ridge | nterm\_c\_NN | 2.255042 |
|  | basic-rep-nter\_core-W10-Ridge | nterm\_c\_IN | 2.224784 |
|  | basic-rep-nter\_core-W10-Ridge | nterm\_c\_II | -4.479827 |

```
<matplotlib.figure.Figure at 0xa920090>
```

In [10]:

```
seqs['seq1'] = df['seq1']
seqs['seq2'] = df['seq2']
N_iter += 1

%run -i 08_aligned_fit_DO_ITER.py
```

|  |  |  | corrR | R2\_score | RMSE | med\_abs\_err | explained\_var | Baysian\_IC | Akaike\_IC | N\_samples | N\_feat |
| --- | --- | --- | --- | --- | --- | --- | --- | --- | --- | --- | --- |
| N\_iter | fit\_type | fit\_class |  |  |  |  |  |  |  |  |  |
| 2 | basic-rep-nter\_core-W10-Ridge | all | 0.822211 | 0.863364 | 8.631356 | 2.467751 | 0.675275 | 798.080597 | 1648.827208 | 172 | 11 |
|  |  | Tm>25 | 0.790986 | 0.764155 | 7.946329 | 2.421499 | 0.546191 | 443.907567 | 848.433226 | 95 | 11 |
|  |  | Tm>55 | 0.777861 | -0.310688 | 5.368823 | 2.227141 | -0.189208 | 76.086676 | 106.003848 | 14 | 11 |
|  |  | on\_target | 0.467796 | 0.156589 | 5.696305 | 2.178260 | 0.162702 | 69.089600 | 93.574507 | 12 | 11 |

|  |  | feature | coef |
| --- | --- | --- | --- |
| N\_iter | fit\_type |  |  |
| 2 | basic-rep-nter\_core-W10-Ridge | c\_NN | -3.840506 |
|  | basic-rep-nter\_core-W10-Ridge | c\_IN | -4.635836 |
|  | basic-rep-nter\_core-W10-Ridge | c\_II | 12.969116 |
|  | basic-rep-nter\_core-W10-Ridge | es\_EE | 0.016273 |
|  | basic-rep-nter\_core-W10-Ridge | es\_EK | 6.705225 |
|  | basic-rep-nter\_core-W10-Ridge | es\_KK | 2.264049 |
|  | basic-rep-nter\_core-W10-Ridge | interface\_repulsion | -0.240818 |
|  | basic-rep-nter\_core-W10-Ridge | nterm\_c\_NN | 2.963611 |
|  | basic-rep-nter\_core-W10-Ridge | nterm\_c\_IN | 1.503457 |
|  | basic-rep-nter\_core-W10-Ridge | nterm\_c\_II | -4.467068 |

In [11]:

```
seqs['seq1'] = df['seq1']
seqs['seq2'] = df['seq2']
N_iter += 1

%run -i 08_aligned_fit_DO_ITER.py
```

|  |  |  | corrR | R2\_score | RMSE | med\_abs\_err | explained\_var | Baysian\_IC | Akaike\_IC | N\_samples | N\_feat |
| --- | --- | --- | --- | --- | --- | --- | --- | --- | --- | --- | --- |
| N\_iter | fit\_type | fit\_class |  |  |  |  |  |  |  |  |  |
| 3 | basic-rep-nter\_core-W10-Ridge | all | 0.824526 | 0.864780 | 8.586518 | 2.445870 | 0.679238 | 796.288944 | 1647.035555 | 172 | 11 |
|  |  | Tm>25 | 0.792816 | 0.766575 | 7.905453 | 2.319299 | 0.554233 | 442.927670 | 847.453329 | 95 | 11 |
|  |  | Tm>55 | 0.777536 | -0.311597 | 5.370685 | 2.219334 | -0.206845 | 76.096383 | 106.013555 | 14 | 11 |
|  |  | on\_target | 0.467152 | 0.157156 | 5.694392 | 2.167270 | 0.162425 | 69.081535 | 93.566442 | 12 | 11 |

|  |  | feature | coef |
| --- | --- | --- | --- |
| N\_iter | fit\_type |  |  |
| 3 | basic-rep-nter\_core-W10-Ridge | c\_NN | -4.886300 |
|  | basic-rep-nter\_core-W10-Ridge | c\_IN | -4.331703 |
|  | basic-rep-nter\_core-W10-Ridge | c\_II | 14.489216 |
|  | basic-rep-nter\_core-W10-Ridge | es\_EE | 0.550005 |
|  | basic-rep-nter\_core-W10-Ridge | es\_EK | 7.248265 |
|  | basic-rep-nter\_core-W10-Ridge | es\_KK | 2.744157 |
|  | basic-rep-nter\_core-W10-Ridge | interface\_repulsion | -0.239183 |
|  | basic-rep-nter\_core-W10-Ridge | nterm\_c\_NN | 3.114699 |
|  | basic-rep-nter\_core-W10-Ridge | nterm\_c\_IN | 1.227372 |
|  | basic-rep-nter\_core-W10-Ridge | nterm\_c\_II | -4.342071 |

In [12]:

```
seqs['seq1'] = df['seq1']
seqs['seq2'] = df['seq2']
N_iter += 1

%run -i 08_aligned_fit_DO_ITER.py
```

|  |  |  | corrR | R2\_score | RMSE | med\_abs\_err | explained\_var | Baysian\_IC | Akaike\_IC | N\_samples | N\_feat |
| --- | --- | --- | --- | --- | --- | --- | --- | --- | --- | --- | --- |
| N\_iter | fit\_type | fit\_class |  |  |  |  |  |  |  |  |  |
| 4 | basic-rep-nter\_core-W10-Ridge | all | 0.824523 | 0.864709 | 8.588792 | 2.482883 | 0.679173 | 796.380033 | 1647.126644 | 172 | 11 |
|  |  | Tm>25 | 0.792392 | 0.766158 | 7.912514 | 2.366830 | 0.552536 | 443.097291 | 847.622950 | 95 | 11 |
|  |  | Tm>55 | 0.776080 | -0.319460 | 5.386757 | 2.225818 | -0.213457 | 76.180052 | 106.097224 | 14 | 11 |
|  |  | on\_target | 0.466343 | 0.155576 | 5.699725 | 2.168181 | 0.161201 | 69.104002 | 93.588909 | 12 | 11 |

|  |  | feature | coef |
| --- | --- | --- | --- |
| N\_iter | fit\_type |  |  |
| 4 | basic-rep-nter\_core-W10-Ridge | c\_NN | -5.170511 |
|  | basic-rep-nter\_core-W10-Ridge | c\_IN | -4.418316 |
|  | basic-rep-nter\_core-W10-Ridge | c\_II | 14.780457 |
|  | basic-rep-nter\_core-W10-Ridge | es\_EE | 0.507674 |
|  | basic-rep-nter\_core-W10-Ridge | es\_EK | 7.215920 |
|  | basic-rep-nter\_core-W10-Ridge | es\_KK | 2.659666 |
|  | basic-rep-nter\_core-W10-Ridge | interface\_repulsion | -0.237809 |
|  | basic-rep-nter\_core-W10-Ridge | nterm\_c\_NN | 3.093885 |
|  | basic-rep-nter\_core-W10-Ridge | nterm\_c\_IN | 1.318748 |
|  | basic-rep-nter\_core-W10-Ridge | nterm\_c\_II | -4.412632 |

In [13]:

```
seqs['seq1'] = df['seq1']
seqs['seq2'] = df['seq2']
N_iter += 1

%run -i 08_aligned_fit_DO_ITER.py
```

|  |  |  | corrR | R2\_score | RMSE | med\_abs\_err | explained\_var | Baysian\_IC | Akaike\_IC | N\_samples | N\_feat |
| --- | --- | --- | --- | --- | --- | --- | --- | --- | --- | --- | --- |
| N\_iter | fit\_type | fit\_class |  |  |  |  |  |  |  |  |  |
| 5 | basic-rep-nter\_core-W10-Ridge | all | 0.825068 | 0.864834 | 8.584828 | 2.466207 | 0.679972 | 796.221223 | 1646.967834 | 172 | 11 |
|  |  | Tm>25 | 0.792287 | 0.765421 | 7.924964 | 2.374511 | 0.549702 | 443.396035 | 847.921694 | 95 | 11 |
|  |  | Tm>55 | 0.774670 | -0.327841 | 5.403840 | 2.250072 | -0.191905 | 76.268707 | 106.185879 | 14 | 11 |
|  |  | on\_target | 0.466790 | 0.150406 | 5.717147 | 2.183545 | 0.156848 | 69.177253 | 93.662159 | 12 | 11 |

|  |  | feature | coef |
| --- | --- | --- | --- |
| N\_iter | fit\_type |  |  |
| 5 | basic-rep-nter\_core-W10-Ridge | c\_NN | -5.353872 |
|  | basic-rep-nter\_core-W10-Ridge | c\_IN | -4.675530 |
|  | basic-rep-nter\_core-W10-Ridge | c\_II | 15.080921 |
|  | basic-rep-nter\_core-W10-Ridge | es\_EE | 0.392022 |
|  | basic-rep-nter\_core-W10-Ridge | es\_EK | 7.091441 |
|  | basic-rep-nter\_core-W10-Ridge | es\_KK | 2.619575 |
|  | basic-rep-nter\_core-W10-Ridge | interface\_repulsion | -0.242089 |
|  | basic-rep-nter\_core-W10-Ridge | nterm\_c\_NN | 2.993196 |
|  | basic-rep-nter\_core-W10-Ridge | nterm\_c\_IN | 1.603522 |
|  | basic-rep-nter\_core-W10-Ridge | nterm\_c\_II | -4.596717 |

In [14]:

```
fit_metrics_all.to_csv('models/{fit_type}.metrics.csv'.format(**locals()) ) 
model_features_all.to_csv('models/{fit_type}.metrics.csv'.format(**locals()) )
```
