# Supplementary material for: A multiplexed bacterial two-hybrid for rapid characterization of protein–protein interactions and iterative protein design
Source: Nat Commun. 2023 Aug 2;14:4636. doi: 10.1038/s41467-023-38697-x (PMC10397247; doi:10.1038/s41467-023-38697-x)
Supplement: Supplementary file 14 — Source Data [file 41467_2023_38697_MOESM14_ESM.zip › Supplementary File S1 Visualization of designed sets.zip!/06_set_visualizations/03_extra_html/MODEL_DNA-ALL-basic-rep-nter_core-Ridge-WsdRD10.html]

DNA\_09\_per\_set\_aligned\_fit\_iterative


In [1]:

```
from __future__ import division, absolute_import, print_function
%load_ext autoreload
%autoreload 2

import os 
print(os.getcwd()) 
    
#import hepran
#import hepran.bzipscore as bz
#import hepran.bcipa as bc
#import hepran.utils as u
#import hepran.registers as r
#import hepran.agadir as ag

import pandas as pd
import numpy as np
import seaborn as sns
    
%matplotlib inline

import matplotlib as mpl
import matplotlib.pylab as plt

import sklearn as sk


CCO = os.environ.get("CCO",r"C:\Projekti_KI\ortoCC\CoiledCoilOrtho")
SFD = os.environ.get("SFD",r"D:\data\ortoCC\design-with-alignments\4heptade-7\!OUT_bcf")
from score_utils import *
```

```
d:\data\ortoCC\fine_tune_scoring_function
```

In [2]:

```
from interactive_set_plot import *

import bokeh
import bokeh.resources
import bokeh.plotting as bp
from bokeh.models import HoverTool
from IPython.core.display import display, HTML
import hepran
bp.output_notebook()

import sklearn
from sklearn import linear_model
from sklearn.externals import joblib

from IPython.display import display, HTML
```

Loading BokehJS ...

In [3]:

```
%%time
df = pd.read_csv('data/DNA_round0_fiting.csv', index_col=[0,1])
df = df.apply(get_CC_features, axis=1)
#df.rename(columns={'RD_Tm':'Tm'}, inplace=True)
df['Tm']=df.RD_Tm
df['norm_sd_RD'] = df.sd_RD/df.sd_RD.mean()
df['cv_RD'] = df.sd_RD/df.mean_RD
```

```
Wall time: 4.41 s
```

In [4]:

```
fit_metrics_all =  get_metrics_df().set_index('N_iter fit_type fit_class'.split())
model_features_all = get_features_df().set_index("N_iter fit_type".split())
```

In [5]:

```
def set_weights(name, df):
    df['weights'] = 1
    if name == "W10L15H55":
        df['weights'] = 1
        df.loc[df.Tm < 15, 'weights'] = 10
        df.loc[df.Tm > 55, 'weights'] = 10
        df.loc[df.on_target == True, 'weights'] = 10
    if name == "W10L15H50":
        df['weights'] = 1
        df.loc[df.Tm < 15, 'weights'] = 10
        df.loc[df.Tm > 50, 'weights'] = 10
        df.loc[df.on_target == True, 'weights'] = 10
    if name == "W10":
        df.loc[df.on_target == True, 'weights'] = 10
    if name == "W10L":
        df['weights'] = 1
        df.loc[df.Tm < 15, 'weights'] = 10
        df.loc[df.on_target == True, 'weights'] = 10
    if name == "WsdRD":
        df['weights'] = 8/df.norm_sd_RD        
    if name == "WsdRD10":
        df['weights'] = 8/df.norm_sd_RD        
        df.loc[df.on_target == True, 'weights'] = 10*df.loc[df.on_target == True, 'weights']
    if name == "WcvRD10":
        df['weights'] = 8/df.cv_RD       
        df.loc[df.on_target == True, 'weights'] = 10*df.loc[df.on_target == True, 'weights']    
    if name == "WcvRD":
        df['weights'] = 8/df.cv_RD       
    if name == "WbnRD10":
        df['weights'] = df.bcnum       
        df.loc[df.on_target == True, 'weights'] = 10*df.loc[df.on_target == True, 'weights']    
    if name == "WbnRD":
        df['weights'] = df.bcnum
```

In [6]:

```
extra_cols="RD_Tm ln_mean_RD bcnum sd_RD norm_sd_RD cv_RD".split()
```

In [7]:

```
fit_type_string = "basic-rep"
lm_type = "Ridge" #Ridge, ElasticNet, SGDRegressor, BayesianRidge
weight_string="WbnRD10" #W1 W10 W10L15H50 W10L15H55
target_field = 'Tm'
set_name = 'ALL'

#read ENV vars if they are set
fit_type_string = os.environ.get('fit_type_string', fit_type_string)
lm_type = os.environ.get('lm_type', lm_type)
weight_string = os.environ.get('weight_string', weight_string)
set_name = os.environ.get('set_name', set_name)

print("fit_type_string =", fit_type_string)
print("lm_type =", lm_type)
print("weight_string =", weight_string)
print("set_name =", set_name)
```

```
fit_type_string = basic-rep-nter_core
lm_type = Ridge
weight_string = WsdRD10
set_name = ALL
```

In [8]:

```
##filter per set
if set_name != "ALL":
    ids = u.get_ids_from_pairs(u.load_set_file(SFD+'/'+set_name))
    df = df.query('(ID1 in @ids) and (ID2 in @ids)')
```

In [9]:

```
#df['weights'] = 1/df.cv_RD**2
#df.plot.scatter("Tm","score")
```

In [10]:

```
tooltips = [
    ('ID1, ID2', '@IDs'),
    ('RD_Tm', '@RD_Tm'),
    ('score', '@score'),
    ('weights', '@weights'),  
    ('ln_mean_RD', '@ln_mean_RD'),
    ('bcnum', '@bcnum'),
    ('sd_RD', '@sd_RD'),    
    ('cv_RD', '@cv_RD'),    
    ('seq1', '@seq1_disp{safe}'),
    ('seq2', '@seq2_disp{safe}'),
]
```

In [11]:

```
fit_type = fit_type_string
fit_fields = fit_fields_dic[fit_type_string]


fit_type = 'DNA-'+set_name.replace('.set','') + "-" +fit_type
set_weights(weight_string, df)


Q=df
N_iter = 0
print("Iteration: ", N_iter)
print("Num points: ", len(Q))
lm, R2 = make_model(target_field, fit_fields, Q, lm_type)
fit_type += "-" + lm_type 
if weight_string:
    fit_type += "-" + weight_string
    
df['score'] = lm.predict(df[fit_fields])
df['pos'] = 0


df = df.apply(get_formated_seq, axis=1)
df['IDs']=df.index
title = str(N_iter)+"_"+fit_type

p = draw_scatter_interactive(target_field, 'score', df, y_range=(0,80),                                
                                 title=title, save_to_file=False, tooltips=tooltips)


bp.show(p)

#mpl_plot_fit(title, df)

fit_metric = get_FIT_dataframe(Q, lm, N_iter, fit_type, N_feat=len(lm.coef_)+1, N_samples=len(Q))
fit_metrics_all = fit_metrics_all.append(fit_metric)
display(fit_metric)
model_features = get_model_features(lm, fit_fields, N_iter, fit_type)
model_features_all = model_features_all.append(model_features)
display(model_features)


joblib.dump(lm, 'models/{title}.model'.format(**locals())); 
df.to_excel('models/{title}.score.xlsx'.format(**locals()));
bp.save(p, title=title, filename='models\\{title}.plot.html'.format(**locals()), resources=bokeh.resources.INLINE);
```

```
Iteration:  0
Num points:  2374
```

|  |  |  | corrR | R2\_score | RMSE | med\_abs\_err | explained\_var | Baysian\_IC | Akaike\_IC | N\_samples | N\_feat |
| --- | --- | --- | --- | --- | --- | --- | --- | --- | --- | --- | --- |
| N\_iter | fit\_type | fit\_class |  |  |  |  |  |  |  |  |  |
| 0 | DNA-ALL-basic-rep-nter\_core-Ridge-WsdRD10 | all | 0.491324 | 0.439365 | 5.783231 | 1.907185 | 0.239621 | 8418.057873 | 26806.077385 | 2374 | 11 |
|  |  | Tm>25 | 0.490615 | 0.440184 | 5.661288 | 1.888661 | 0.237983 | 8164.105800 | 26166.758804 | 2330 | 11 |
|  |  | Tm>55 | 0.267138 | -7.143431 | 18.749927 | 4.666512 | -0.193441 | 420.940017 | 663.360820 | 64 | 11 |
|  |  | on\_target | 0.569817 | 0.094676 | 7.619424 | 2.331994 | 0.184622 | 531.722922 | 1064.186177 | 118 | 11 |

|  |  | feature | coef |
| --- | --- | --- | --- |
| N\_iter | fit\_type |  |  |
| 0 | DNA-ALL-basic-rep-nter\_core-Ridge-WsdRD10 | c\_NN | 0.217064 |
|  | DNA-ALL-basic-rep-nter\_core-Ridge-WsdRD10 | c\_IN | -1.703817 |
|  | DNA-ALL-basic-rep-nter\_core-Ridge-WsdRD10 | c\_II | 1.486753 |
|  | DNA-ALL-basic-rep-nter\_core-Ridge-WsdRD10 | es\_EE | -0.915554 |
|  | DNA-ALL-basic-rep-nter\_core-Ridge-WsdRD10 | es\_EK | 0.889689 |
|  | DNA-ALL-basic-rep-nter\_core-Ridge-WsdRD10 | es\_KK | 0.025865 |
|  | DNA-ALL-basic-rep-nter\_core-Ridge-WsdRD10 | interface\_repulsion | -0.172565 |
|  | DNA-ALL-basic-rep-nter\_core-Ridge-WsdRD10 | nterm\_c\_NN | 0.381037 |
|  | DNA-ALL-basic-rep-nter\_core-Ridge-WsdRD10 | nterm\_c\_IN | 0.177070 |
|  | DNA-ALL-basic-rep-nter\_core-Ridge-WsdRD10 | nterm\_c\_II | -0.558107 |

In [12]:

```
N_iter = 1
seqs = df

%run -i 08_aligned_fit_DO_ITER.py
```

|  |  |  | corrR | R2\_score | RMSE | med\_abs\_err | explained\_var | Baysian\_IC | Akaike\_IC | N\_samples | N\_feat |
| --- | --- | --- | --- | --- | --- | --- | --- | --- | --- | --- | --- |
| N\_iter | fit\_type | fit\_class |  |  |  |  |  |  |  |  |  |
| 1 | DNA-ALL-basic-rep-nter\_core-Ridge-WsdRD10 | all | 0.533122 | 0.478659 | 5.576878 | 1.837418 | 0.284057 | 8245.546520 | 26633.566032 | 2374 | 11 |
|  |  | Tm>25 | 0.533140 | 0.480161 | 5.455403 | 1.814693 | 0.284227 | 7991.476497 | 25994.129502 | 2330 | 11 |
|  |  | Tm>55 | 0.306279 | -6.784407 | 18.331949 | 4.683917 | -0.214279 | 418.054320 | 660.475123 | 64 | 11 |
|  |  | on\_target | 0.529832 | 0.123737 | 7.496132 | 2.322697 | 0.154863 | 527.872885 | 1060.336140 | 118 | 11 |

|  |  | feature | coef |
| --- | --- | --- | --- |
| N\_iter | fit\_type |  |  |
| 1 | DNA-ALL-basic-rep-nter\_core-Ridge-WsdRD10 | c\_NN | 0.981754 |
|  | DNA-ALL-basic-rep-nter\_core-Ridge-WsdRD10 | c\_IN | -1.771113 |
|  | DNA-ALL-basic-rep-nter\_core-Ridge-WsdRD10 | c\_II | 2.519188 |
|  | DNA-ALL-basic-rep-nter\_core-Ridge-WsdRD10 | es\_EE | 0.263739 |
|  | DNA-ALL-basic-rep-nter\_core-Ridge-WsdRD10 | es\_EK | 2.282288 |
|  | DNA-ALL-basic-rep-nter\_core-Ridge-WsdRD10 | es\_KK | 0.913632 |
|  | DNA-ALL-basic-rep-nter\_core-Ridge-WsdRD10 | interface\_repulsion | -0.144187 |
|  | DNA-ALL-basic-rep-nter\_core-Ridge-WsdRD10 | nterm\_c\_NN | 0.131068 |
|  | DNA-ALL-basic-rep-nter\_core-Ridge-WsdRD10 | nterm\_c\_IN | 0.662498 |
|  | DNA-ALL-basic-rep-nter\_core-Ridge-WsdRD10 | nterm\_c\_II | -0.793566 |

In [13]:

```
seqs['seq1'] = df['seq1']
seqs['seq2'] = df['seq2']
N_iter += 1

%run -i 08_aligned_fit_DO_ITER.py
```

|  |  |  | corrR | R2\_score | RMSE | med\_abs\_err | explained\_var | Baysian\_IC | Akaike\_IC | N\_samples | N\_feat |
| --- | --- | --- | --- | --- | --- | --- | --- | --- | --- | --- | --- |
| N\_iter | fit\_type | fit\_class |  |  |  |  |  |  |  |  |  |
| 2 | DNA-ALL-basic-rep-nter\_core-Ridge-WsdRD10 | all | 0.545493 | 0.489055 | 5.520994 | 1.835697 | 0.297096 | 8197.728763 | 26585.748275 | 2374 | 11 |
|  |  | Tm>25 | 0.544296 | 0.489253 | 5.407484 | 1.818177 | 0.296123 | 7950.363140 | 25953.016144 | 2330 | 11 |
|  |  | Tm>55 | 0.286116 | -6.604201 | 18.118517 | 4.662993 | -0.258759 | 416.555327 | 658.976131 | 64 | 11 |
|  |  | on\_target | 0.532914 | 0.134538 | 7.449792 | 2.352411 | 0.159046 | 526.409444 | 1058.872699 | 118 | 11 |

|  |  | feature | coef |
| --- | --- | --- | --- |
| N\_iter | fit\_type |  |  |
| 2 | DNA-ALL-basic-rep-nter\_core-Ridge-WsdRD10 | c\_NN | 1.246929 |
|  | DNA-ALL-basic-rep-nter\_core-Ridge-WsdRD10 | c\_IN | -1.855419 |
|  | DNA-ALL-basic-rep-nter\_core-Ridge-WsdRD10 | c\_II | 2.700070 |
|  | DNA-ALL-basic-rep-nter\_core-Ridge-WsdRD10 | es\_EE | 0.470799 |
|  | DNA-ALL-basic-rep-nter\_core-Ridge-WsdRD10 | es\_EK | 2.522052 |
|  | DNA-ALL-basic-rep-nter\_core-Ridge-WsdRD10 | es\_KK | 1.190309 |
|  | DNA-ALL-basic-rep-nter\_core-Ridge-WsdRD10 | interface\_repulsion | -0.148217 |
|  | DNA-ALL-basic-rep-nter\_core-Ridge-WsdRD10 | nterm\_c\_NN | -0.083050 |
|  | DNA-ALL-basic-rep-nter\_core-Ridge-WsdRD10 | nterm\_c\_IN | 0.961751 |
|  | DNA-ALL-basic-rep-nter\_core-Ridge-WsdRD10 | nterm\_c\_II | -0.878700 |

In [14]:

```
seqs['seq1'] = df['seq1']
seqs['seq2'] = df['seq2']
N_iter += 1

%run -i 08_aligned_fit_DO_ITER.py
```

|  |  |  | corrR | R2\_score | RMSE | med\_abs\_err | explained\_var | Baysian\_IC | Akaike\_IC | N\_samples | N\_feat |
| --- | --- | --- | --- | --- | --- | --- | --- | --- | --- | --- | --- |
| N\_iter | fit\_type | fit\_class |  |  |  |  |  |  |  |  |  |
| 3 | DNA-ALL-basic-rep-nter\_core-Ridge-WsdRD10 | all | 0.545399 | 0.489276 | 5.519803 | 1.835066 | 0.297010 | 8196.703852 | 26584.723364 | 2374 | 11 |
|  |  | Tm>25 | 0.544233 | 0.489530 | 5.406023 | 1.815174 | 0.296063 | 7949.103332 | 25951.756336 | 2330 | 11 |
|  |  | Tm>55 | 0.284673 | -6.589296 | 18.100752 | 4.658690 | -0.263221 | 416.429759 | 658.850563 | 64 | 11 |
|  |  | on\_target | 0.536273 | 0.136132 | 7.442926 | 2.351957 | 0.160414 | 526.191835 | 1058.655089 | 118 | 11 |

|  |  | feature | coef |
| --- | --- | --- | --- |
| N\_iter | fit\_type |  |  |
| 3 | DNA-ALL-basic-rep-nter\_core-Ridge-WsdRD10 | c\_NN | 1.294791 |
|  | DNA-ALL-basic-rep-nter\_core-Ridge-WsdRD10 | c\_IN | -1.819985 |
|  | DNA-ALL-basic-rep-nter\_core-Ridge-WsdRD10 | c\_II | 2.675226 |
|  | DNA-ALL-basic-rep-nter\_core-Ridge-WsdRD10 | es\_EE | 0.501802 |
|  | DNA-ALL-basic-rep-nter\_core-Ridge-WsdRD10 | es\_EK | 2.556499 |
|  | DNA-ALL-basic-rep-nter\_core-Ridge-WsdRD10 | es\_KK | 1.241765 |
|  | DNA-ALL-basic-rep-nter\_core-Ridge-WsdRD10 | interface\_repulsion | -0.148647 |
|  | DNA-ALL-basic-rep-nter\_core-Ridge-WsdRD10 | nterm\_c\_NN | -0.086614 |
|  | DNA-ALL-basic-rep-nter\_core-Ridge-WsdRD10 | nterm\_c\_IN | 0.920853 |
|  | DNA-ALL-basic-rep-nter\_core-Ridge-WsdRD10 | nterm\_c\_II | -0.834239 |

In [15]:

```
seqs['seq1'] = df['seq1']
seqs['seq2'] = df['seq2']
N_iter += 1

%run -i 08_aligned_fit_DO_ITER.py
```

|  |  |  | corrR | R2\_score | RMSE | med\_abs\_err | explained\_var | Baysian\_IC | Akaike\_IC | N\_samples | N\_feat |
| --- | --- | --- | --- | --- | --- | --- | --- | --- | --- | --- | --- |
| N\_iter | fit\_type | fit\_class |  |  |  |  |  |  |  |  |  |
| 4 | DNA-ALL-basic-rep-nter\_core-Ridge-WsdRD10 | all | 0.545312 | 0.489328 | 5.519521 | 1.840923 | 0.296936 | 8196.461584 | 26584.481096 | 2374 | 11 |
|  |  | Tm>25 | 0.544107 | 0.489545 | 5.405940 | 1.814919 | 0.295937 | 7949.031937 | 25951.684941 | 2330 | 11 |
|  |  | Tm>55 | 0.283975 | -6.581407 | 18.091342 | 4.658660 | -0.266334 | 416.363197 | 658.784001 | 64 | 11 |
|  |  | on\_target | 0.538172 | 0.137064 | 7.438912 | 2.349680 | 0.161622 | 526.064527 | 1058.527782 | 118 | 11 |

|  |  | feature | coef |
| --- | --- | --- | --- |
| N\_iter | fit\_type |  |  |
| 4 | DNA-ALL-basic-rep-nter\_core-Ridge-WsdRD10 | c\_NN | 1.324202 |
|  | DNA-ALL-basic-rep-nter\_core-Ridge-WsdRD10 | c\_IN | -1.813619 |
|  | DNA-ALL-basic-rep-nter\_core-Ridge-WsdRD10 | c\_II | 2.676156 |
|  | DNA-ALL-basic-rep-nter\_core-Ridge-WsdRD10 | es\_EE | 0.538003 |
|  | DNA-ALL-basic-rep-nter\_core-Ridge-WsdRD10 | es\_EK | 2.573929 |
|  | DNA-ALL-basic-rep-nter\_core-Ridge-WsdRD10 | es\_KK | 1.261547 |
|  | DNA-ALL-basic-rep-nter\_core-Ridge-WsdRD10 | interface\_repulsion | -0.149685 |
|  | DNA-ALL-basic-rep-nter\_core-Ridge-WsdRD10 | nterm\_c\_NN | -0.102104 |
|  | DNA-ALL-basic-rep-nter\_core-Ridge-WsdRD10 | nterm\_c\_IN | 0.931368 |
|  | DNA-ALL-basic-rep-nter\_core-Ridge-WsdRD10 | nterm\_c\_II | -0.829264 |

In [16]:

```
seqs['seq1'] = df['seq1']
seqs['seq2'] = df['seq2']
N_iter += 1

%run -i 08_aligned_fit_DO_ITER.py
```

|  |  |  | corrR | R2\_score | RMSE | med\_abs\_err | explained\_var | Baysian\_IC | Akaike\_IC | N\_samples | N\_feat |
| --- | --- | --- | --- | --- | --- | --- | --- | --- | --- | --- | --- |
| N\_iter | fit\_type | fit\_class |  |  |  |  |  |  |  |  |  |
| 5 | DNA-ALL-basic-rep-nter\_core-Ridge-WsdRD10 | all | 0.545297 | 0.489329 | 5.519517 | 1.841029 | 0.296921 | 8196.457988 | 26584.477501 | 2374 | 11 |
|  |  | Tm>25 | 0.544091 | 0.489545 | 5.405939 | 1.814624 | 0.295920 | 7949.030903 | 25951.683907 | 2330 | 11 |
|  |  | Tm>55 | 0.283967 | -6.581043 | 18.090908 | 4.658629 | -0.266463 | 416.360129 | 658.780932 | 64 | 11 |
|  |  | on\_target | 0.538302 | 0.137103 | 7.438743 | 2.349864 | 0.161681 | 526.059169 | 1058.522424 | 118 | 11 |

|  |  | feature | coef |
| --- | --- | --- | --- |
| N\_iter | fit\_type |  |  |
| 5 | DNA-ALL-basic-rep-nter\_core-Ridge-WsdRD10 | c\_NN | 1.324978 |
|  | DNA-ALL-basic-rep-nter\_core-Ridge-WsdRD10 | c\_IN | -1.813056 |
|  | DNA-ALL-basic-rep-nter\_core-Ridge-WsdRD10 | c\_II | 2.675831 |
|  | DNA-ALL-basic-rep-nter\_core-Ridge-WsdRD10 | es\_EE | 0.539000 |
|  | DNA-ALL-basic-rep-nter\_core-Ridge-WsdRD10 | es\_EK | 2.574382 |
|  | DNA-ALL-basic-rep-nter\_core-Ridge-WsdRD10 | es\_KK | 1.262125 |
|  | DNA-ALL-basic-rep-nter\_core-Ridge-WsdRD10 | interface\_repulsion | -0.149716 |
|  | DNA-ALL-basic-rep-nter\_core-Ridge-WsdRD10 | nterm\_c\_NN | -0.100730 |
|  | DNA-ALL-basic-rep-nter\_core-Ridge-WsdRD10 | nterm\_c\_IN | 0.929704 |
|  | DNA-ALL-basic-rep-nter\_core-Ridge-WsdRD10 | nterm\_c\_II | -0.828975 |

In [17]:

```
fit_metrics_all.to_csv('models/{fit_type}.metrics.csv'.format(**locals()) ) 
model_features_all.to_csv('models/{fit_type}.features.csv'.format(**locals()) )
```

In [18]:

```
fit_type
```

Out[18]:

```
'DNA-ALL-basic-rep-nter_core-Ridge-WsdRD10'
```
