# Supplementary material for: A multiplexed bacterial two-hybrid for rapid characterization of protein–protein interactions and iterative protein design
Source: Nat Commun. 2023 Aug 2;14:4636. doi: 10.1038/s41467-023-38697-x (PMC10397247; doi:10.1038/s41467-023-38697-x)
Supplement: Supplementary file 14 — Source Data [file 41467_2023_38697_MOESM14_ESM.zip › Supplementary File S1 Visualization of designed sets.zip!/06_set_visualizations/03_extra_html/SCORE_DNA-ALL-basic-rep-nter_core-Ridge-WsdRD10___PNIC-mason.bA.set.html]

DNA\_10\_scoring\_function\_validation


In [1]:

```
from __future__ import division, absolute_import, print_function
%load_ext autoreload
%autoreload 2

import os 
print(os.getcwd()) 
    
#import hepran
#import hepran.bzipscore as bz
#import hepran.bcipa as bc
#import hepran.utils as u
#import hepran.registers as r
#import hepran.agadir as ag

import pandas as pd
import numpy as np
import seaborn as sns
    
%matplotlib inline

import matplotlib as mpl
import matplotlib.pylab as plt

import sklearn as sk


CCO = os.environ.get("CCO",r"C:\Projekti_KI\ortoCC\CoiledCoilOrtho")
SFD = os.environ.get("SFD",r"D:\data\ortoCC\fine_tune_scoring_function\sets")
from score_utils import *
from set_utils import *
```

```
d:\data\ortoCC\fine_tune_scoring_function
```

In [2]:

```
from interactive_set_plot import *

import bokeh
import bokeh.resources
import bokeh.plotting as bp
from bokeh.models import HoverTool
from IPython.core.display import display, HTML
import hepran
bp.output_notebook()

import sklearn
from sklearn import linear_model
from sklearn.externals import joblib

from IPython.display import display, HTML
```

Loading BokehJS ...

In [3]:

```
%%time
df = pd.read_csv('data/DNA_round0_fiting.csv', index_col=[0,1])
```

```
Wall time: 83 ms
```

In [4]:

```
extra_cols="RD_Tm ln_mean_RD bcnum sd_RD norm_sd_RD cv_RD".split()
fit_metrics_all =  get_metrics_df().set_index('N_iter fit_type fit_class'.split())
fit_metrics_all['OI_score'] = ''
fit_metrics_all['GAP_score'] = ''
fit_metrics_all['NAM_score'] = ''
fit_metrics_all['OI_exp'] = ''
fit_metrics_all['GAP_exp'] = ''
fit_metrics_all['NAM_exp'] = ''
```

In [5]:

```
target_field = 'Tm'
set_name = 'PNIC.bA.set'
fit_name = 'DNA-ALL-basicL-rep-Ridge-WbnRD10'

#read ENV vars if they are set
set_name = os.environ.get('set_name', set_name)
fit_name = os.environ.get('fit_name', fit_name)
```

In [6]:

```
##filter per set
if set_name != "ALL":
    pairs = u.load_set_file(SFD+'/'+set_name)
    ids = u.get_ids_from_pairs(pairs)
    df = df.query('(ID1 in @ids) and (ID2 in @ids)')
```

In [7]:

```
tooltips = [
    ('ID1, ID2', '@IDs'),
    ('RD_Tm', '@RD_Tm'),
    ('score', '@score'),
    ('weights', '@weights'),  
    ('ln_mean_RD', '@ln_mean_RD'),
    ('bcnum', '@bcnum'),
    ('sd_RD', '@sd_RD'),    
    ('cv_RD', '@cv_RD'),    
    ('seq1', '@seq1_disp{safe}'),
    ('seq2', '@seq2_disp{safe}'),
]
```

In [8]:

```
df = df.apply(get_CC_features, axis=1)
#df.rename(columns={'RD_Tm':'Tm'}, inplace=True)
df['Tm']=df.RD_Tm
df['norm_sd_RD'] = df.sd_RD/df.sd_RD.mean()
df['cv_RD'] = df.sd_RD/df.mean_RD
df['weights'] = 1
```

In [9]:

In [9]:

```
def do_plots(aiter, do_score_overlap=False):
    #load model 
    global fit_metrics_all
    global df
    global N_iter
    global lm
    global fit_fields
    global model_features_all
    N_iter = aiter
    model_features_all = pd.read_csv('models/{fit_name}.features.csv'.format(**globals()), index_col=[0,1])
    fit_fields = model_features_all.loc[N_iter]['feature'].values
    lm = joblib.load('models/{N_iter}_{fit_name}.model'.format(**globals()))
    if not do_score_overlap:
        df['score'] = lm.predict(df[fit_fields])
        df['pos'] = 0
        Q = df
    else:
        Q = score_overlap(df, lm, fit_fields, get_CC_features)
    
    
    Q = Q.apply(get_formated_seq, axis=1)
    Q['IDs']=Q.index
    title = fit_name
    #bp.output_file(title=title, filename='models\\{title}.plot.html'.format(**locals()))
    p = draw_scatter_interactive(target_field, 'score', Q, y_range=(0,80),                                
                                     title=title, save_to_file=False, tooltips=tooltips, width=450, height=450)


    
    ##show model table
    from bokeh.models.widgets import DataTable, NumberFormatter, TableColumn
    columns = [
            TableColumn(field="feature", title="Feature"),
            TableColumn(field="coef", title="Coeficients", formatter=NumberFormatter(format='+0.000')),
        ]
    model_feat = DataTable(source=bp.ColumnDataSource(model_features_all.loc[N_iter]),
                                                columns = columns,
                                                width=400, height=450)

    bp.show(
        bokeh.layouts.row(
            p,
            bokeh.layouts.widgetbox(model_feat),

        ))
    
    
    fit_metric = get_FIT_dataframe(Q, lm, N_iter, fit_name, N_feat=len(lm.coef_)+1, N_samples=len(Q))

    
    

    fig, axs = plt.subplots(1,2,figsize=[12,8])
    ax, OI, GAP, NAM = plot_orto_set(Q, ids, title=title, field="RD_Tm", pairs=pairs, ax=axs[0])
    fit_metric['OI_exp'] = OI
    fit_metric['GAP_exp'] = GAP
    fit_metric['NAM_exp'] = NAM
    
    ax, OI, GAP, NAM = plot_orto_set(Q, ids, title=title, field="score", pairs=pairs, ax=axs[1])
    fit_metric['OI_mod'] = OI
    fit_metric['GAP_mod'] = GAP
    fit_metric['NAM_mod'] = NAM
    
    
    fit_metric['set'] = set_name
    fit_metrics_all = fit_metrics_all.append(fit_metric)
    display(fit_metric)
    
do_plots(0, do_score_overlap=False)
```

|  |  |  | corrR | R2\_score | RMSE | med\_abs\_err | explained\_var | Baysian\_IC | Akaike\_IC | N\_samples | N\_feat | OI\_exp | GAP\_exp | NAM\_exp | OI\_mod | GAP\_mod | NAM\_mod | set |
| --- | --- | --- | --- | --- | --- | --- | --- | --- | --- | --- | --- | --- | --- | --- | --- | --- | --- | --- |
| N\_iter | fit\_type | fit\_class |  |  |  |  |  |  |  |  |  |  |  |  |  |  |  |  |
| 0 | DNA-ALL-basic-rep-nter\_core-Ridge-WsdRD10 | all | 0.587178 | 0.215667 | 8.345497 | 2.220664 | 0.343494 | 967.122796 | 2078.415927 | 214 | 11 | 42.784258 | -18.857159 | 12 | 30.245966 | 0.581839 | 0 | PNIC-mason.bA.set |
|  |  | Tm>25 | 0.589481 | 0.205044 | 8.358991 | 2.179680 | 0.345862 | 954.918945 | 2047.290571 | 211 | 11 | 42.784258 | -18.857159 | 12 | 30.245966 | 0.581839 | 0 | PNIC-mason.bA.set |
|  |  | Tm>55 | 0.031350 | -9.928243 | 21.421872 | 4.167786 | -0.569848 | 107.889167 | 135.019066 | 13 | 11 | 42.784258 | -18.857159 | 12 | 30.245966 | 0.581839 | 0 | PNIC-mason.bA.set |
|  |  | on\_target | 0.239575 | -2.605007 | 16.110899 | 3.144298 | 0.054009 | 106.855519 | 136.772691 | 14 | 11 | 42.784258 | -18.857159 | 12 | 30.245966 | 0.581839 | 0 | PNIC-mason.bA.set |

In [10]:

```
do_plots(1, do_score_overlap=True)
```

|  |  |  | corrR | R2\_score | RMSE | med\_abs\_err | explained\_var | Baysian\_IC | Akaike\_IC | N\_samples | N\_feat | OI\_exp | GAP\_exp | NAM\_exp | OI\_mod | GAP\_mod | NAM\_mod | set |
| --- | --- | --- | --- | --- | --- | --- | --- | --- | --- | --- | --- | --- | --- | --- | --- | --- | --- | --- |
| N\_iter | fit\_type | fit\_class |  |  |  |  |  |  |  |  |  |  |  |  |  |  |  |  |
| 1 | DNA-ALL-basic-rep-nter\_core-Ridge-WsdRD10 | all | 0.681241 | 0.338971 | 7.661471 | 2.092668 | 0.453940 | 930.521060 | 2041.814191 | 214 | 11 | 42.784258 | -18.857159 | 12 | 30.692079 | 3.239331 | 0 | PNIC-mason.bA.set |
|  |  | Tm>25 | 0.681744 | 0.329436 | 7.677190 | 2.078476 | 0.455700 | 919.013477 | 2011.385104 | 211 | 11 | 42.784258 | -18.857159 | 12 | 30.692079 | 3.239331 | 0 | PNIC-mason.bA.set |
|  |  | Tm>55 | 0.168958 | -8.687050 | 20.168705 | 4.131039 | -0.145906 | 106.321879 | 133.451778 | 13 | 11 | 42.784258 | -18.857159 | 12 | 30.692079 | 3.239331 | 0 | PNIC-mason.bA.set |
|  |  | on\_target | 0.232401 | -2.390292 | 15.623750 | 3.150905 | 0.053728 | 105.995812 | 135.912984 | 14 | 11 | 42.784258 | -18.857159 | 12 | 30.692079 | 3.239331 | 0 | PNIC-mason.bA.set |

In [11]:

```
do_plots(2, do_score_overlap=True)
```

|  |  |  | corrR | R2\_score | RMSE | med\_abs\_err | explained\_var | Baysian\_IC | Akaike\_IC | N\_samples | N\_feat | OI\_exp | GAP\_exp | NAM\_exp | OI\_mod | GAP\_mod | NAM\_mod | set |
| --- | --- | --- | --- | --- | --- | --- | --- | --- | --- | --- | --- | --- | --- | --- | --- | --- | --- | --- |
| N\_iter | fit\_type | fit\_class |  |  |  |  |  |  |  |  |  |  |  |  |  |  |  |  |
| 2 | DNA-ALL-basic-rep-nter\_core-Ridge-WsdRD10 | all | 0.687773 | 0.330840 | 7.708443 | 2.104691 | 0.464675 | 933.137103 | 2044.430234 | 214 | 11 | 42.784258 | -18.857159 | 12 | 31.090541 | 3.598876 | 0 | PNIC-mason.bA.set |
|  |  | Tm>25 | 0.688739 | 0.320766 | 7.726662 | 2.078634 | 0.466930 | 921.724105 | 2014.095732 | 211 | 11 | 42.784258 | -18.857159 | 12 | 31.090541 | 3.598876 | 0 | PNIC-mason.bA.set |
|  |  | Tm>55 | 0.158981 | -8.518567 | 19.992543 | 4.092326 | -0.170161 | 106.093786 | 133.223685 | 13 | 11 | 42.784258 | -18.857159 | 12 | 31.090541 | 3.598876 | 0 | PNIC-mason.bA.set |
|  |  | on\_target | 0.240113 | -2.297007 | 15.407304 | 3.085704 | 0.057410 | 105.605199 | 135.522371 | 14 | 11 | 42.784258 | -18.857159 | 12 | 31.090541 | 3.598876 | 0 | PNIC-mason.bA.set |

In [12]:

```
do_plots(3, do_score_overlap=True)
```

|  |  |  | corrR | R2\_score | RMSE | med\_abs\_err | explained\_var | Baysian\_IC | Akaike\_IC | N\_samples | N\_feat | OI\_exp | GAP\_exp | NAM\_exp | OI\_mod | GAP\_mod | NAM\_mod | set |
| --- | --- | --- | --- | --- | --- | --- | --- | --- | --- | --- | --- | --- | --- | --- | --- | --- | --- | --- |
| N\_iter | fit\_type | fit\_class |  |  |  |  |  |  |  |  |  |  |  |  |  |  |  |  |
| 3 | DNA-ALL-basic-rep-nter\_core-Ridge-WsdRD10 | all | 0.687848 | 0.331496 | 7.704669 | 2.108925 | 0.464892 | 932.927467 | 2044.220598 | 214 | 11 | 42.784258 | -18.857159 | 12 | 31.117677 | 3.612878 | 0 | PNIC-mason.bA.set |
|  |  | Tm>25 | 0.688757 | 0.321403 | 7.723042 | 2.086841 | 0.467067 | 921.526344 | 2013.897970 | 211 | 11 | 42.784258 | -18.857159 | 12 | 31.117677 | 3.612878 | 0 | PNIC-mason.bA.set |
|  |  | Tm>55 | 0.157209 | -8.493955 | 19.966679 | 4.082776 | -0.172999 | 106.060129 | 133.190027 | 13 | 11 | 42.784258 | -18.857159 | 12 | 31.117677 | 3.612878 | 0 | PNIC-mason.bA.set |
|  |  | on\_target | 0.242552 | -2.286050 | 15.381681 | 3.075758 | 0.058639 | 105.558594 | 135.475766 | 14 | 11 | 42.784258 | -18.857159 | 12 | 31.117677 | 3.612878 | 0 | PNIC-mason.bA.set |

In [13]:

```
do_plots(4, do_score_overlap=True)
```

|  |  |  | corrR | R2\_score | RMSE | med\_abs\_err | explained\_var | Baysian\_IC | Akaike\_IC | N\_samples | N\_feat | OI\_exp | GAP\_exp | NAM\_exp | OI\_mod | GAP\_mod | NAM\_mod | set |
| --- | --- | --- | --- | --- | --- | --- | --- | --- | --- | --- | --- | --- | --- | --- | --- | --- | --- | --- |
| N\_iter | fit\_type | fit\_class |  |  |  |  |  |  |  |  |  |  |  |  |  |  |  |  |
| 4 | DNA-ALL-basic-rep-nter\_core-Ridge-WsdRD10 | all | 0.687631 | 0.331824 | 7.702776 | 2.103689 | 0.464624 | 932.822329 | 2044.115460 | 214 | 11 | 42.784258 | -18.857159 | 12 | 31.132551 | 3.574491 | 0 | PNIC-mason.bA.set |
|  |  | Tm>25 | 0.688600 | 0.321807 | 7.720740 | 2.100082 | 0.466871 | 921.400556 | 2013.772182 | 211 | 11 | 42.784258 | -18.857159 | 12 | 31.132551 | 3.574491 | 0 | PNIC-mason.bA.set |
|  |  | Tm>55 | 0.155695 | -8.472989 | 19.944620 | 4.076366 | -0.175038 | 106.031388 | 133.161287 | 13 | 11 | 42.784258 | -18.857159 | 12 | 31.132551 | 3.574491 | 0 | PNIC-mason.bA.set |
|  |  | on\_target | 0.243776 | -2.279650 | 15.366695 | 3.067411 | 0.059219 | 105.531300 | 135.448472 | 14 | 11 | 42.784258 | -18.857159 | 12 | 31.132551 | 3.574491 | 0 | PNIC-mason.bA.set |

In [14]:

```
do_plots(5, do_score_overlap=True)
```

|  |  |  | corrR | R2\_score | RMSE | med\_abs\_err | explained\_var | Baysian\_IC | Akaike\_IC | N\_samples | N\_feat | OI\_exp | GAP\_exp | NAM\_exp | OI\_mod | GAP\_mod | NAM\_mod | set |
| --- | --- | --- | --- | --- | --- | --- | --- | --- | --- | --- | --- | --- | --- | --- | --- | --- | --- | --- |
| N\_iter | fit\_type | fit\_class |  |  |  |  |  |  |  |  |  |  |  |  |  |  |  |  |
| 5 | DNA-ALL-basic-rep-nter\_core-Ridge-WsdRD10 | all | 0.687617 | 0.331823 | 7.702780 | 2.103886 | 0.464612 | 932.822533 | 2044.115664 | 214 | 11 | 42.784258 | -18.857159 | 12 | 31.133394 | 3.57158 | 0 | PNIC-mason.bA.set |
|  |  | Tm>25 | 0.688586 | 0.321806 | 7.720749 | 2.100845 | 0.466856 | 921.401048 | 2013.772675 | 211 | 11 | 42.784258 | -18.857159 | 12 | 31.133394 | 3.57158 | 0 | PNIC-mason.bA.set |
|  |  | Tm>55 | 0.155641 | -8.472229 | 19.943819 | 4.076201 | -0.175109 | 106.030344 | 133.160243 | 13 | 11 | 42.784258 | -18.857159 | 12 | 31.133394 | 3.57158 | 0 | PNIC-mason.bA.set |
|  |  | on\_target | 0.243732 | -2.279384 | 15.366071 | 3.067249 | 0.059194 | 105.530164 | 135.447336 | 14 | 11 | 42.784258 | -18.857159 | 12 | 31.133394 | 3.57158 | 0 | PNIC-mason.bA.set |

In [15]:

```
out_name = "SCORE_" + fit_name + "___" + set_name
fit_metrics_all.to_csv('models/{out_name}.metrics.csv'.format(**locals()) )
```
