# Supplementary material for: A multiplexed bacterial two-hybrid for rapid characterization of protein–protein interactions and iterative protein design
Source: Nat Commun. 2023 Aug 2;14:4636. doi: 10.1038/s41467-023-38697-x (PMC10397247; doi:10.1038/s41467-023-38697-x)
Supplement: Supplementary file 14 — Source Data [file 41467_2023_38697_MOESM14_ESM.zip › Supplementary File S1 Visualization of designed sets.zip!/06_set_visualizations/0_no_shifting_DNA-ALL-basic-rep-nter_core-Ridge-WsdRD10.plot.html]

0\_DNA-ALL-basic-rep-nter\_core-Ridge-WsdRD10
